# Host Variability in NTM Disease: Implications for Research Needs

**DOI:** 10.3389/fmicb.2018.02901

**Published:** 2018-12-03

**Authors:** Colin Swenson, Christa S. Zerbe, Kevin Fennelly

**Affiliations:** ^1^Division of Pulmonary, Allergy, Critical Care, and Sleep Medicine, Emory University, Atlanta, GA, United States; ^2^Laboratory of Clinical Immunology and Microbiology, National Institute of Allergy and Infectious Diseases, National Institutes of Health, Bethesda, MD, United States; ^3^Laboratory of Chronic Airway Infection, Pulmonary Branch, Division of Intramural Research, National Heart, Lung, and Blood Institute, National Institutes of Health, Bethesda, MD, United States

**Keywords:** non-tuberculous mycobacteria, pulmonary, bronchiectasis, disseminated, COPD, cystic fibrosis, osteomyelitis

## Abstract

Non-tuberculous mycobacteria (NTM) are ubiquitous environmental organisms that may cause opportunistic infections in susceptible hosts. Lung infections in immunocompetent persons with structural lung disease are most common, while disseminated disease occurs primarily in immunocompromised individuals. Human disease caused by certain species, such as *Mycobacterium avium* complex, *Mycobacterium abscessus*, and *Mycobacterium kansasii*, is increasing in incidence and varies by geographic distribution. The spectrum of NTM disease varies widely in presentation and clinical outcome, but certain patterns can be organized into clinical phenotypes. Treatment options are limited, lengthy, and often toxic. The purpose of this case-based review is to provide non-clinician scientists with a better understanding of human NTM disease with an aim to stimulate more research and development.

“It is more important to know what sort of person has a disease than to know what sort of disease a person has.”

– Hippocrates, Greek Physician, 460–370 B.C.

## Introduction

Non-tuberculous mycobacteria (NTM) are ubiquitous environmental organisms that are frequently isolated from soil and water, as well as other environmental sources. NTM are acid-fast bacilli (AFB) that are clinically categorized as either rapid growers (RG), taking fewer than 7 days to grow on culture media, or slow growers (SG), taking more than 7 days (Table 1). Certain species are well known causes of human disease, most commonly *Mycobacterium avium* complex (MAC), which includes the species *M. avium*, *M. intracellulare*, and *M. chimaera*, and the *M. abscessus* complex (MABSC), which includes the subspecies *abscessus*, *massiliense*, and *bolletii*. Considered opportunistic pathogens, NTM organisms may occasionally be isolated from the sputa samples of healthy individuals, but there is a higher prevalence of NTM infection among patients with cystic fibrosis and non-cystic fibrosis bronchiectasis, as well as chronic obstructive pulmonary disease (COPD) (Adjemian et al., 2012; Fleshner et al., 2016). While all MAC species may be isolated from water and soil sources, *M. avium* is more commonly isolated from fresh water sources, and *M*. *intracellulare* is associated with garden soils (Falkinham, 2015). This predilection has been proposed as an explanation for the epidemiologic clusters observed in regions with abundant surface water and loamy soil.

**Table 1:** Clinically important mycobacterial species organized by growth time.

Slow growing mycobacteria	Rapid growing mycobacteria
*M. avium complex^∗^*	*M. abscessus complex^∗∗^*
*M. kansasii*	*M. fortuitum*
*M. xenopi*	*M. chelonae*
*M. szulgai*	*M. mucogenicum*
*M. simiae*	


*Slow growing organisms take greater than 7 days to culture. Rapid growing organisms take fewer than 7 days to culture. ^∗^Includes M. avium, M. intracellulare, and M. chimaera. ^∗∗^Includes M. abscessus, M. massiliense, and M. bolletii.*

While NTM species may rarely cause pulmonary infections in normal hosts, those with structural lung disease, such as bronchiectasis and emphysema, are at increased risk. The hallmark of the disease is granulomatous inflammation of the airways, which, over time, may lead to worsening bronchiectasis and cavitary lung disease (Fujita et al., 1999). Among patients with pulmonary NTM infections, distinct phenotypes have been described based primarily on the underlying lung disease. These phenotypes have characteristic presentations on lung imaging (Table 2).

**Table 2:** Radiographic presentations of pulmonary NTM diseases.

Radiographic appearance	Representative underlying disease
Fibrocavitary	Chronic obstructive pulmonary disease (COPD)
Nodular bronchiectasis	Idiopathic bronchiectasis in thin older women
Upper lobe bronchiectasis	Cystic fibrosis in children and young adults
Lower lobe bronchiectasis and chronic sinusitis	Primary ciliary dyskinesia in children, young adults
Lower lobe bronchiectasis	Chronic GERD and aspiration in older adult
Upper/mid-lung fine nodules	Hypersensitivity pneumonitis: ‘hot-tub lung’


Although once considered rare diseases, human infections with NTM appear to be increasing in North America and Europe, and they are increasingly recognized in areas of the world considered endemic for tuberculosis (TB), including Africa, South America and India (Adjemian et al., 2012). Human lung disease caused by NTM was first recognized in the 1950s as the incidence of TB was decreasing (Timpe and Runyon, 1954). Given the similarities of clinical microbiology and of the drugs used to treat TB, the care of patients with NTM disease has often been provided by clinicians and hospitals initially established to manage TB. The HIV/AIDS epidemic introduced many physicians and scientists to disseminated infections with MAC, which has waned in incidence along with other opportunistic infections due to the development and widespread use of highly active antiretroviral therapy. Most disseminated NTM infections occur in immunocompromised patients. Similar to TB, pulmonary NTM disease accounts for approximately 85% of cases and disseminated NTM disease for about 15% (Jones et al., 2018). The major types of human NTM disease are summarized in Table 3.

**Table 3:** Types of NTM disease based on underlying pathophysiology.

Type of NTM disease	Underlying pathophysiology
***Pulmonary***	
COPD	Protease-antiprotease imbalance
Cystic fibrosis (CF) bronchiectasis	CFTR abnormalities: abnormal airway surface fluid
Non-CF bronchiectasis: child/young adult	Primary ciliary dyskinesia: abnormal mucociliary clearance
Non-CF bronchiectasis	Idiopathic; Chronic aspiration; Chest wall abnormalities: scoliosis, pectus excavatum, etc. Primary immunodeficiencies, e.g., CVID Connective tissue diseases, e.g., RA, Sjogren’s syndrome
Hypersensitivity pneumonitis	Exposures to aerosolized NTM; immunological
***Cervical lymphadenitis***	Children via oropharyngeal mucosa inoculation
***Skin and soft tissue infections***	Direct inoculation; surgical wound infections
***Osteoarticular infections***	Trauma
***Disseminated infection***	HIV/AIDS; Contaminated heart-lung machine in cardiac surgery; Immunological deficiencies in the Th1 pathway: *Interferon gamma receptor; Interleukin-12 receptor; Signal transducer and activator of transcription 1 (STAT1); Interferon regulatory factor 8; GATA2 (MonoMAC syndrome); Interferon-stimulated gene 15; Nuclear factor-kappa-B essential modulator (NEMO); X-linked chronic granulomatous disease*


The primary purpose of this review is to introduce microbiologists and other scientists who are not clinicians to the wide variety of clinical presentations among humans infected with NTM. Given the variable responses observed to infections with the same pathogen among various inbred strains of mice and other experimental animal models, it should not be surprising that the ‘human host’ is not just one host, but rather many. However, in spite of the enormous genetic variability among humans, there are similarities in presentations of NTM disease among various human ‘phenotypes.’ We will present representative cases, most of which are actual cases that we have seen, followed by a discussion of that category of disease and figures of imaging studies. We hope that a better understanding of the various human diseases caused by NTM can help inspire our non-clinical colleagues to develop better diagnostic tests, drug therapies and vaccines and other preventive interventions.

## Protection of Research Participants

Written consent was obtained from each patient presented in this article. He or she explicitly consented to the inclusion of background and clinical data, as well as radiographic images.

## Non-Cystic Fibrosis Bronchiectasis (NCFB)

### Case

**FIGURE 1:**
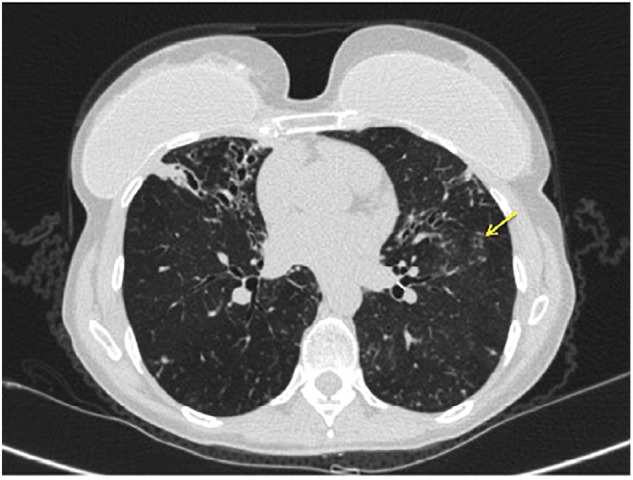
Axial CT chest image demonstrating bronchiectasis and mucoid impaction in the right middle lobe. The tree-in-bud nodularity in the left lung (yellow arrow).

A 67-year-old female lifelong non-smoker with a history of scoliosis and early osteoporosis developed a non-productive cough for 6 months, an unintentional weight loss, and daytime fatigue. She had experienced a seven-pound weight loss over 6 months, to a body mass index (BMI) of 17.5 kg/m^2^ (normal: 19–24 kg/m^2^). An avid swimmer and jogger, she had also noticed a gradual decline in her stamina, with an increase in shortness of breath with moderate exercise. Her primary care provider ordered a chest radiograph, which showed possible right middle lobe pneumonia. She was prescribed 10 days of levofloxacin, which did not improve her respiratory symptoms. She developed severe diarrhea, and on the seventh day of levofloxacin, her husband took her to the emergency department, where she was admitted for dehydration secondary to *Clostridium difficile* colitis. Because the cough had not improved with antibiotics, a CT of the chest was performed, showing right middle lobe and lingula bronchiectasis, nodularity, and mucus impaction (Figure 1). She was placed on airborne isolation for possible pulmonary tuberculosis. MTB PCR probe was negative on an induced sputum sample, so she was taken for bronchoscopy with lavage. These samples were smear positive for AFB, and 10 days later, grew *M. avium*. She was discharged from the hospital on a combination of ethambutol, rifampin, and azithromycin three times weekly, and after 3 months of treatment, her cough resolved and she began to gain weight. Repeat CT imaging at 6 months demonstrated fewer lung nodules, with less mucus impaction.

### Discussion

The nodular-bronchiectatic phenotype of pulmonary NTM disease is also known as “Lady Windermere Syndrome,” in reference to the character from Oscar Wilde’s 1892 play entitled “Lady Windermere’s Fan” (Reich and Johnson, 1992). While the term has had wide adoption in the medical literature, it is, in fact, a malapropism, since the character for which the syndrome is named is a young woman without respiratory symptoms of any kind. Further, the authors who originally named this syndrome believed that the infection resulted from women chronically suppressing their cough, leading to mucus inspissation in the longer airways of the right middle lobe and lingula. This hypothesis has more recently been challenged, since a hallmark of the nodular-bronchiectatic phenotype is chronic cough and sputum production (Olivier, 2016; Tsai et al., 2017). Reflex cough responses in elderly women with stable pulmonary NTM disease appear similar to age-matched controls, except for a mildly decreased urge-to-cough, arguing against cough suppression (Tsai et al., 2017).

Individuals with this phenotype are typically female, elderly, are tall with thin body habitus, have pectus excavatum, scoliosis, and a non-smoking history (Kim et al., 2008; Park et al., 2009; Lee et al., 2013). Radiographically, the right middle lobe and lingula are most often affected, though any area of the lung may be affected (Figure 1). Typically, the radiographic pattern is described as “tree-in-bud,” indicating bronchiolar inflammation and mucus impaction. Coinfection with more than one species of *Mycobacterium*, or with Gram-negative bacteria, characterizes later, more advanced disease.

Symptomatically, patients with the nodular-bronchiectatic phenotype typically complain of chronic cough, which may or may not be productive, and may or may not be associated with hemoptysis, persistent fatigue, night sweats, and unintentional weight loss. The infection may be mistaken for active pulmonary tuberculosis, leading to airborne isolation and prolonged hospitalization, as happened with our patient. Treatment is typically a prolonged combination regimen of a rifamycin, most often rifampin, ethambutol, and a macrolide, typically azithromycin or clarithromycin. The frequency of dosing depends on the extent of radiographic disease: mild nodular-bronchiectatic disease may be treated with a thrice-weekly regimen, while more severe bronchiectasis requires a daily regimen. In the most severe cases, particularly those with cystic bronchiectasis and cavitary lung disease, a concurrent thrice-weekly parenteral aminoglycoside, such as amikacin, may be necessary (Griffith et al., 2007). As with the *M. abscessus* group, surgical resection in localized or cavitary disease is an option where medical treatment has failed to eradicate infection. Since individuals with the nodular-bronchiectatic phenotype are typically elderly and frail, the risks of surgical resection may outweigh potential benefits, in which case a chronic suppressive strategy with antibiotics is a reasonable alternative.

Complete clearance of MAC infection is variable, and often depends on the severity of disease. Further limiting the successful clearance of MAC infection is the low rate of clinician adherence to the 2007 ATS/IDSA treatment guidelines (Adjemian et al., 2014a,b). With the guidelines-based treatment approach, more than 60% of patients with nodular-bronchiectatic disease will have treatment success (Wallace et al., 2014; Diel et al., 2018). These patients continue to be susceptible to reinfection, however, often with entirely new strains or subspecies. Overall, the recurrence of pulmonary MAC infection approaches 40%, with the highest rates of recurrence among the nodular-bronchiectatic cohort (Lee et al., 2015; Boyle et al., 2016). The rate of relapsed disease, where the same clinical isolate is again isolated within 12 months of supposed cure, is unknown. While environmental exposure has been proposed as a source of infection, it is unclear what role remediation and exposure avoidance have on preventing reinfection.

## Cystic Fibrosis Lung Disease (CF)

### Case

**FIGURE 2:**
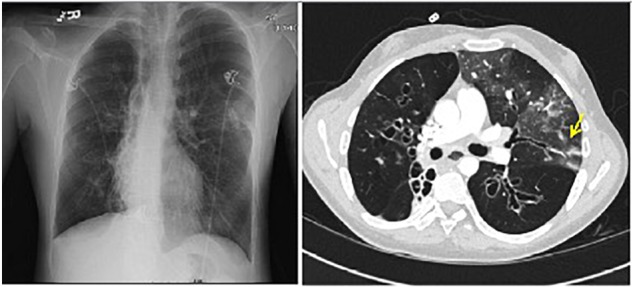
Left: chest radiograph demonstrating cystic bronchiectasis. Right: CT image of chest showing a left upper lobe infiltrate on a background of cystic bronchiectasis (yellow arrow).

A 21-year-old man with CF presented to the outpatient clinic with worsening dyspnea, weight loss, fever, and hemoptysis over approximately 2 months. He had last been in clinic 3 months prior when he had reported feeling well. Induced sputum sample during that clinic visit grew scant mucoid fluoroquinolone-resistant *Pseudomonas aeruginosa*. Mycobacterial cultures were negative at that time, and his forced expiratory volume (FEV_1_: typically the best overall measure of lung function) was stable at 2.5 L (normal for patient’s age: 4.5 L). His BMI was chronically low at 17.8 kg/m2 (normal: 19–24 kg/m^2^). At the current clinic visit, his FEV_1_ had declined to 2.1 L, and his sputum sample was a dark gray streaked with blood. Because his resting pulse oximetry reading was consistently in the upper-80% range, he was directly admitted to hospital, where sputum sample was smear positive for AFB. *M. tuberculosis* PCR probe was negative. Chest radiograph on admission demonstrated upper and mid-lung zone cystic bronchiectasis with a new possible left upper lobe infiltrate (Figure 2, left). CT of chest confirmed the development of a left upper lobe infiltrate, with new tree-in-bud attenuation (Figure 2, right). The sputum culture grew *M. abscessus* 5 days after admission.

### Discussion

Patients with CF have a higher prevalence of NTM disease than the general population. Pathogenic NTM species are isolated from approximately 1 in 5 patients with CF, and the incidence appears to be increasing by 5% per year, depending on geographic location (Adjemian et al., 2014a, 2018). Among the NTM species affecting this patient population, approximately 60% of isolates are MAC, while almost 40% are MABSC. MABSC is comprised of three subspecies: *M. abscessus*, *M. bolletii*, and *M*. *massiliense*, all of which are rapidly growing organisms. MABSC is a notoriously difficult infection to treat, owing in part to the *erm*(41) gene, which confers inducible macrolide resistance (Adjemian et al., 2018). The subspecies *M. massiliense* is an exception, however, since it lacks a functional *erm*(41) gene, and is therefore more easily eradicated (Kim et al., 2010; Roux et al., 2015). Patients with CF who develop MABSC infection typically have a lower BMI and, paradoxically, a higher FEV_1_ than those without MABSC infection (Adjemian et al., 2018).

Infection with MABSC is considered a relative contraindication to lung transplantation, since MABSC infection may prove fatal in the post-transplant period, when recipients are on maximal immunosuppressive therapy (Chalermskulrat et al., 2006; Gilljam et al., 2010). Because subjects with CF have progressive lung disease, lung transplantation is often a consideration at the later stages of disease. Our patient was concurrently followed by the lung transplant clinic, though he had never been listed. His sputum isolate was ultimately identified as subspecies *M. massiliense*, and he was successfully treated with a regimen consisting of azithromycin, amikacin, and cefoxitin. Sputum samples at 3, 6, and 9 months were negative for NTM species. His hemoptysis resolved, and he did not require supplemental oxygen at his three-month follow-up appointment.

## Chronic Obstructive Lung Disease (COPD)

### Case

**FIGURE 3:**
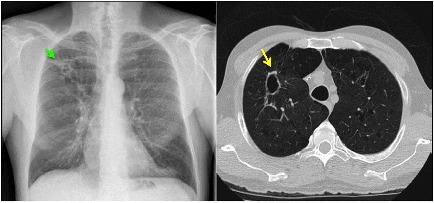
Left: chest radiograph demonstrating a right upper lobe cavitary lesion on a background of emphysema (green arrow). Right: axial CT image demonstrating the same cavitary lesion in the right lung (yellow arrow). Reprinted with permission of the American Thoracic Society. Copyright© 2018 American Thoracic Society. Fennelly et al. (2016) is an official journal of the American Thoracic Society.

**FIGURE 4:**
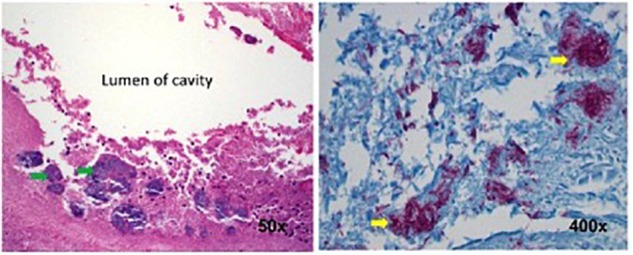
Histopathology of the surgical specimen (using hematoxylin and eosin stains) demonstrating aggregates of bacteria (left, green arrows) along the inner wall of the lung cavity, with many AFB seen at higher power using Ziehl Nissen stain (right, yellow arrows). Reprinted with permission of the American Thoracic Society. Copyright© 2018 American Thoracic Society. Fennelly et al. (2016) is an official journal of the American Thoracic Society.

A 55-year-old man with a history of COPD associated with smoking developed a chronic cough productive of sputum, malaise, and night sweats. The social history was remarkable for use of a hot tub at his home. Sputum microbiology was notable for 3+ AFB on smear and three cultures grew *M. abscessus*, susceptible to amikacin and linezolid, and intermediate to clarithromycin (without *erm* gene testing) and cefoxitin. Pulmonary function testing showed airflow obstruction and a low diffusing capacity, and imaging demonstrated a right upper lobe cavity and emphysematous changes, consistent with COPD (Figure 3). He and his wife had just retired and sold their home in northern Florida. They were en route to vacation in the American West in their recreational vehicle (RV), hoping to receive some oral antibiotics and be on their way. His symptoms responded well to treatment with intravenous amikacin once daily and intravenous imipenem-cilastatin twice daily and azithromycin (home health nursing visited him in a state forest campground where they lived in their RV during his treatment). However, 4 months after treatment he was admitted to the hospital with new shortness of breath, fevers, and with ground-glass opacities, consistent with parenchymal inflammation, in the posterior aspects of both upper lobes. Bronchoalveolar lavage (BAL) grew only *M. abscessus*, and the BAL cell count was markedly lymphocytic. This pattern was consistent with an allergic alveolitis due to a spill of the cavity, so he was treated with oral corticosteroids and his antimycobacterial regimen intensified by the addition of linezolid once daily. As the cavity was the major site of disease and his lung function was only mildly decreased, he was referred for surgical resection of the right upper lobe. The histopathology of the cavity was remarkable for aggregates of AFB along the inner wall of the cavity (Figure 4). *M. abscessus* grew from a culture of the resected lung cavity within 3 days. Scanning electron microscopy of the inner wall of the resected cavity demonstrated bacilli embedded within a matrix. There were 3.83 × 10^7^ colony forming units (CFU) of total bacteria in a 0.5 g sample from the lung cavity; 7.17 × 10^5^ CFU were in biofilm. The bacterial composition was 97.7% mycobacterial using 16s ribosomal RNA sequencing (Fennelly et al., 2016). The patient’s symptoms and imaging improved post-operatively, and all sputa specimens were negative on culture in the subsequent 10 months of his tour of the West.

### Discussion

Patients with COPD often have emphysema that is typically more prominent in the upper lobes. The ‘blebs’ or holes within the lung parenchyma are presumably infected by NTM. The mode of infection is not well understood, but we suspect that it was likely due to inhalation in this case. We see a similar pattern of *Aspergillus fumigatus* infecting the upper lobes of COPD patients, and *Aspergillus* in the most ubiquitous airborne mold. As TB often presents with upper lobe cavities, COPD patients with AFB in their sputum are often initially placed in respiratory isolation as TB suspects unless a rapidly done molecular diagnostic can confirm that the AFB are not *M. tuberculosis*.

**FIGURE 5:**
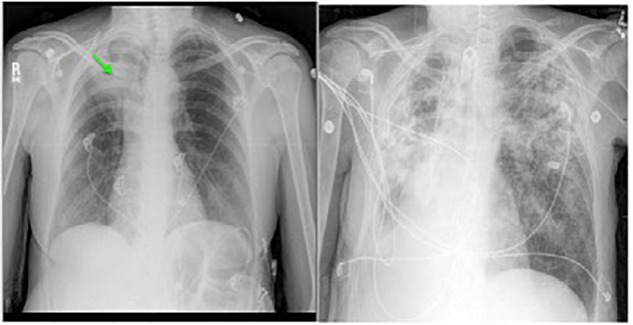
Left: chest radiograph showing right upper lobe consolidation with central cavitary changes (green arrow) in a woman with COPD and pulmonary NTM disease. Right: chest radiograph of same subject demonstrating marked progression of pulmonary NTM disease, including likely spillage of the previous right upper lobe cavitary lesion.

Pulmonary NTM infections in COPD patients have a low cure rate, generally considered to be about 50% compared to about 75% in patients with idiopathic bronchiectasis (Jones et al., 2018). Whether or not this lower cure rate is due to the presence of cavities is unknown. Surgical resection of a cavity can be curative, as in this case. Unfortunately, many patients with cavitary disease have lung function that it so diminished that they cannot tolerate surgery. Surgical resection may also be contraindicated if there is extensive bilateral disease. However, medical treatment can have a role in preventing further progression of disease, possibly by suppressing bacterial growth within cavities or by protecting healthy lung tissue by eradicating small amounts of bacteria that are spread from diseased areas. Figure 5 shows the chest radiographs of a woman with COPD who had cavitary pulmonary MAC disease (Figure 5, Left). Although her sputum remained AFB smear- and culture-positive during her course of treatment, her symptoms had improved and there was no further radiographic evidence of disease progression. Unfortunately, she stopped all her medications when she was given a diagnosis cervical cancer and became depressed, and she was lost to follow-up. Approximately 6 months later she was admitted to the intensive care unit, and a repeat chest radiograph showed dramatic progression of disease (Figure 5, Right). The right upper lobe cavity appeared to have extended and spilled, destroying her entire right lung and significantly involving the left. She died 2 weeks later. COPD appears to be the most important underlying condition leading to mortality in patients with pulmonary NTM infections (Hayashi et al., 2012; Ito et al., 2012; Mirsaeidi et al., 2014; Gommans et al., 2015; Jones et al., 2018), although fibrocavitary disease without COPD is also associated with increased mortality (Fleshner et al., 2016).

## Primary Ciliary Dyskinesia (PCD)

### Case

A 53 year old woman is followed for bronchiectasis, sinusitis and chronic lung infections with MAC and *P. aeruginosa*. She had no respiratory distress as an infant, but she began to develop recurrent sinus infection and a productive cough around age four. At the age of 18 she was diagnosed with bronchiectasis. She had been evaluated for CF with sweat chloride tests were negative on three occasions. She has two children, and no other family members have respiratory disease. On exam now, she is thin with a body-mass index of 17.9 (normal: 19–24 kg/m^2^). On lung exam she has scattered wheezes and rales in the bases. She inhales nebulized hypertonic saline twice daily and engages in aerobic exercise daily for airway clearance.

**FIGURE 6:**
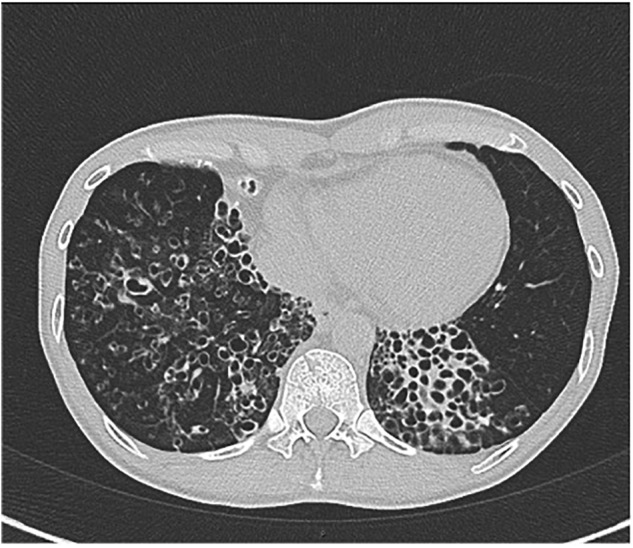
CT chest image showing cystic bronchiectasis in the right and left lower lobes.

Imaging of her sinuses shows chronic sinusitis, with air-fluid levels in both maxillary sinuses and opacified ethmoid and frontal sinuses, in spite of evidence of prior surgeries. Computed tomography (CT) of the chest is remarkable for severe bronchiectasis, predominantly in both lower lobes (Figure 6).

At the age of 37, she was first diagnosed with pulmonary MAC infection after presenting with worsening cough. She improved on treatment with clarithromycin, ethambutol and rifampin for 9 months. However, her cough returned several months after treatment was stopped, and she was restarted on the same treatment except with azithromycin instead of clarithromycin. Unfortunately, she noticed difficulties discriminating red and green colors and was diagnosed with optic neuritis secondary to ethambutol but this resolved after discontinuation of the drug. She was treated with inhaled amikacin but it was stopped due to severe hoarseness. Linezolid was recently added but the discontinued after she developed numbness and tingling in her fingers and toes consistent with peripheral neuropathy.

At the age of 47, the diagnosis of PCD was confirmed by low nasal nitric oxide of 15 nL/min and genetic analysis demonstrating two disease-causing mutations in *DNAH11*. Electron microscopy showed no structural changes of the cilia as seen in classic PCD.

*P. aeruginosa* was first cultured from her sputum in early 2017, but she was feeling relatively well. She received three courses of oral ciprofloxacin without eradication. In the fall of 2017, she complained of more cough and sputum production. She was treated with cefepime and tobramycin by the intravenous route for 2 weeks to treat a bronchiectasis exacerbation due to *P. aeruginosa*. She had baseline mild tinnitus that did not change. A follow-up audiological evaluation 1 month later showed the development of mild to moderate high frequency sensorineural hearing loss bilaterally, although the patient did not perceive hearing loss. Follow-up audiological evaluations after another month and then 6 months later showed no improvement in the hearing acuity, but it remained stable. Unfortunately, her *Pseudomonas* infection now appears chronic. She is feeling relatively well on suppressive therapy with inhaled aztreonam.

### Discussion

PCD, or immotile cilia syndrome, is an autosomal recessive disorder of motile cilia dysfunction resulting in impairment of mucociliary clearance. It is heterogenous, with over 30 genetic variants described to date and a wide range of clinical manifestations. Unlike the patient above, approximately 80% of newborns have neonatal respiratory distress. About one-half of PCD patients have ‘laterality defects’ such as situs inversus, in which the organs are on the opposite side of the body due to defects in the embryonic nodal cilia. In children, CF and PCD may both present with respiratory infections and distress. In CF, the problem is due to alterations of the airway surface fluid, whereas in PCD the problem is with poor movement of the fluid due to the dysfunctional cilia. Both result in bronchiectasis (dilated, chronically inflamed bronchi), but the location tends to be in the upper lobes in CF and in the lower lobes in PCD. Nearly all PCD patients have rhinosinusitis. Infertility is a possibility, but not universal as in this case. The median age of diagnosis in childhood is about 5 years of age, but the age of diagnosis in adults is widely variable. Diagnosis has traditionally been difficult due to variable availability and accuracy of tests such as transmission emission microscopy to evaluate ‘classic’ ciliary ultrastructural defects and nasal nitric oxide measurements. However, the increased availability of genetic testing has been a significant advance. A recent clinical practice guideline provides a very helpful diagnostic algorithm (Shapiro et al., 2018).

Most PCD patients live an active life and have a normal lifespan, although lung infections can interfere with their normal social functions. The rate of decline in their lung function is slower than in CF. The rates of NTM infections among patients with PCD is not known. In a recent registry study, PCD patients were less likely to have NTM infections than those with bronchiectasis associated with gastroesophageal reflux disease (GERD).

This case highlights problems of drug toxicity experienced by most patients with NTM lung diseases, who are also frequently co-infected with Gram-negative bacteria or fungal pathogens. In review, this patient has had adverse reactions to ethambutol (optic neuritis), inhaled amikacin (laryngitis), linezolid (peripheral neuropathy), and intravenous tobramycin (ototoxicity). Due to the multiple adverse reactions, her chronic MAC infection now can be treated only with azithromycin and rifampin, a suboptimal regimen for cure, which is probably unlikely to occur due to the severity of her bronchiectasis. The goal of therapy for both the MAC and the *Pseudomonas* is now suppression of bacterial growth to prevent exacerbations and further lung destruction. Such adverse reactions to drugs limit the armamentarium available to physicians to treat patients, and contribute to the urgent need for additional antibacterial drugs.

## Hypersensitivity Pneumonitis (HP)

### Case

**FIGURE 7:**
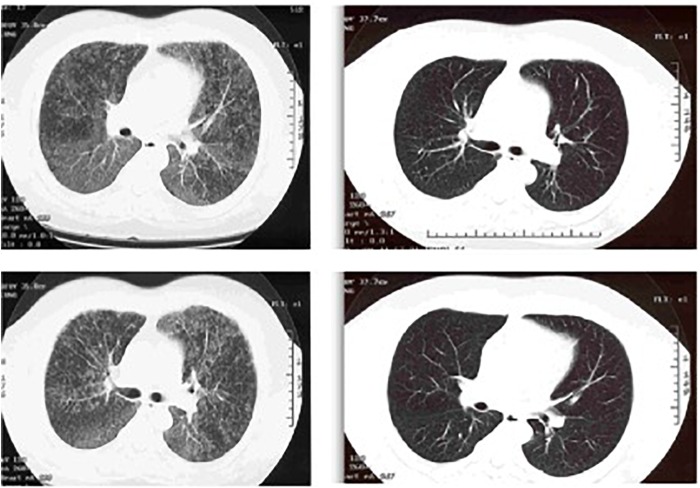
Left: CT chest showing diffuse ground glass centrilobular nodules and mosaic attenuation. Right: post-treatment images demonstrating resolution of the prior findings.

One of us (KF) was consulted on the case of a 52 year-old pulp mill worker for evaluation of possible underlying occupational lung disease. He had been referred to the infectious disease service after a surgical lung biopsy at an outside hospital showed non-caseating granulomas and AFB. The lung tissue culture had grown MAC. The patient had noted progressive shortness of breath. On physical examination, the patient had a very rapid breathing rate in spite of being on supplemental oxygen by nasal cannula with a reservoir device at 15 l/min. There were inspiratory crackles on lung exam. The lung imaging was remarkable for diffuse ‘mosaic attenuation’ and fine nodules (Figure 7) which was not consistent with what is usually seen in MAC infections. It was originally suspected that he might have HP due to airborne molds within the pulp mill. However, the history revealed that he had not worked inside the pulp mill for several years due to a severe back injury. He worked in a control room in another building at a distance from the plant. To manage his back pain, the patient had the habit of soaking in a hot tub for 30 min in the morning after awakening and then again in the evening when he returned from work. The hot tub was in a room just outside his bedroom. He was too short of breath to be able to do pulmonary function testing. We considered performing a bronchoscopy to obtain BAL fluid for cell counts, but we realized that both HP and mycobacterial infection would be predominantly lymphocytic. Given his tenuous respiratory status and his tissue diagnosis, the risk of the procedure far outweighed any benefit. He was treated with both systemic corticosteroids for the HP and with azithromycin, rifampin and ethambutol for the MAC infection. He began to improve within a few days in the hospital, and was discharged home with the advice to drain the hot tub after obtaining a water sample for culture. He sent the water to the local state public health laboratory and received a report that it grew MAC. Unfortunately, when we called to request the isolate to determine if it was identical to the clinical one, the laboratory in a southeastern state had already discarded it because they were overwhelmed with such cultures. When we saw him 18 months after his removal from exposure and initiation of treatment, his symptoms, exam, imaging and pulmonary function tests were completely normal.

### Discussion

This was a classic case of ‘hot tub lung’ due to inhalation of NTM. This form of HP was first described in case reports in 1997, and multiple cases have now been reported in the literature. In a retrospective case series of 21 patients at a referral center, all patients presented with shortness of breath and cough and about half were hypoxemic. MAC was isolated from both the hot tub water and the respiratory secretions or tissue in all cases (Hanak et al., 2006). All improved with avoidance of exposure, and there was a mix of additional corticosteroid and antimycobacterial therapy, but five (24%) received neither. Thus, avoidance of exposure appears to be the most important element. However, in patients with severe illness like our case, more aggressive treatment is warranted. Some of the more interesting case reports have involved multiple immunocompetent family members developing HP due to MAC and possibly other NTM (Mangione et al., 2001; Kitahara et al., 2016).

An occupational respiratory illness associated with NTM that emerged in the last decade is HP associated with metal-working fluids in both the United States and the United Kingdom (Dawkins et al., 2006). These fluids were found to be contaminated with a rapidly growing *Mycobacterium* now known as *M. immunogenum* (Wallace et al., 2002).

*Mycobacterium chelonae* was isolated from a bassoon played by a professional musician diagnosed with HP, but molds were also isolated and no NTM or molds were isolated from the BAL from the patient (Moller et al., 2017). Thus, the association may not be causal. NTM contamination in a water-damaged building was recently associated with asthma symptoms (Park et al., 2017). Environmental sampling for NTM is not commonly done in indoor environments, suggesting a need for further research about environmental exposures to airborne NTM.

## Disseminated Disease

### Case

A 49-year-old denied any infections or hospitalizations in his childhood. As a young adult he had histoplasmosis lymphadenitis and was treated successfully. He did well until 47 years of age when he presented to a hospital complaining of diarrhea after having several year history of GERD, intermittent nausea and emesis. Colonoscopy was performed and pathology showed Periodic Acid Schiff stain (PAS) positivity, so empiric treatment for Whipple’s disease, a rare systemic infection caused by the bacterium *Tropheryma whipplei*, was initiated. One month later, he presented to a large tertiary hospital with mycobacteremia, hypotension and sepsis. He was diagnosed with idiopathic CD4 lymphopenia and disseminated MAC. He suffered multiple cerebral embolic events, had a tracheostomy placed, underwent PEG placement and suffered extensive end-organ damage from long term aminoglycoside therapy. His GI disease was indicative of his overall burden of disease. He was referred to the NIH after the diagnoses of two variants (IL12Rβ1 c.512 A > C, p.Q171P; and IL12Rβ1 c. 1442A > G, p.Y481C) were found in IL-12 receptor 1. He required years of intensive multidrug therapy in addition to immune therapies including exogenous interferon gamma.

### Discussion

Genetic susceptibility to disseminated mycobacterial infection is well described and should be considered in anyone who presents with disseminated disease without HIV infection (Wu and Holland, 2015). Even diseases that are often diagnosed in childhood can present with disseminated infection in adulthood when their mutation results in a less severe phenotype (Hsu et al., 2018). Host control depends on the interleukin 12-interferon pathway that connects myeloid and lymphoid cells. The genetic susceptibilities, if inherited, can be elucidated by a careful family medical history. Autosomal dominant patterns of inheritance include: IFNR1/R2, STAT1, IRF8, GATA2, IL12Rβ1/R2 X-linked: NEMO. Autosomal recessive patterns of inheritance typically present early in life and would not have been considered in this age patient. Non-HIV Acquired immunodeficiency such as autoantibodies to interferon gamma (IFN) would have been another consideration in this patient and one that is important to consider in adults with disseminated infection. However, in the United States, women have been identified more frequently than men, and have mainly been described in the East Asian population.

IL-12Rβ1 encodes the 1 chain of IL-12 and IL-23 receptors and activation of pSTAT4 and is important in the subsequent signaling of IFN and activation of macrophage intracellular killing. IL-12Rβ1 has been reported in more than 200 patients worldwide (de Beaucoudrey et al., 2010). Typically, they will present after BCG vaccination in countries where TB is endemic. However, in the United States, these patients may present later in life, as our patient presented. This case of a primary immune deficiency that phenotypically presented in adulthood highlights the importance of evaluating genetic disorders that may predispose individuals to disseminated infection with a low virulent organism in the absence of known risk factors.

### Case

A 32-year-old Filipina woman presented to her primary care provider with fevers, neck and back pain, and cervical lymphadenopathy. She was diagnosed with community-acquired pneumonia, and treated with azithromycin and oral corticosteroids as well as a subsequent course of doxycycline with more oral corticosteroids. While her pain improved on the corticosteroids, she started having night sweats off of prednisone. An eventual CT of her neck showed destruction of her C3 vertebra, and lymphadenopathy. A PET/CT showed uptake in activity in lymph nodes, femurs, left acetabulum, left pubic ramus, sacrum, right ischium, both iliac bones, ribs, and clavicles. She underwent C3 corpectomy with fusion; followed by right femoral curettage 1 month later. When AFB+ organisms were seen, an interferon gamma release assay (IGRA) was indeterminate. Cultures grew MAC. Three months after the first surgery, she continued to have pain and underwent debridement of an epidural abscess in T8, involving a laminectomy, foraminectomy and hardware placement of screws at T6, T7, T10, and T11. Her disease continued to worsen despite optimal multi-drug therapy. Subsequent testing at the National Institutes of Health (NIH) showed very high titer autoantibodies to Interferon gamma. She was referred to the NIH and continued on multi-drug antibiotic therapy and given rituximab with improvement in her disease. Unfortunately, when her B-cells returned after 2 years, her disease recurred and she remains on therapy for her infection and is getting repeat rituximab infusions for the IFN autoantibodies.

### Discussion

Autoantibodies to IFN were first reported by Doffinger et al. (2004). An adult-onset immunodeficiency due to these autoantibodies in Thailand and Taiwan was described in 2012 in a population of HIV negative patients presenting with opportunistic infections (Browne et al., 2012). They have since been described in patients with thymic neoplasia (Burbelo et al., 2010). An indeterminate IGRA test is characteristic in these patients, as sera from them will block positive controls for this commercial test and will therefore be reported as indeterminate. *In vitro*, the pSTAT1 activation is negative when patient sera is tested in the presence of normal PBMCs. Especially in Asians, one should consider autoantibodies to IFN when encountering an adult with disseminated NTM, particularly when there is no family history of disease.

## Vertebral Osteomyelitis

### Case

**FIGURE 8:**
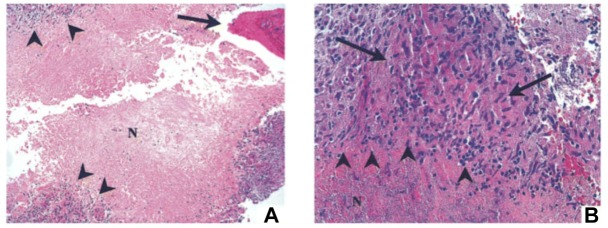
Bone biopsy specimen (T9 vertebra) of patient 1 showing necrotizing granulomas. **(A)** Area of necrotic bone is shown (arrow; hematoxylin and eosin stain; original magnification, ×100). Surrounding the area of caseous necrosis (N) is a rim of granulomatous tissue reaction (arrowheads). **(B)** At higher magnification (hematoxylin and eosin stain; magnification, ×400), the granulomas are principally composed of macrophages (area between the arrows) and lymphocytes (arrowheads). Acid-fast and fungal stains showed negative results, but culture was positive for *Mycobacterium abscessus*. Reprinted with permission of Oxford University Press; Chan et al. (2001).

A 16-year-old girl was evaluated for progressive mid-thoracic back pain. She reported the onset after abatement of symptoms of a flu-like illness with fever, nasal congestion, sore throat and non-productive cough and muscle aches. She was a competitive roller-skater, and she had experienced multiple falls in the prior year. She recalled multiple ecchymoses and abrasions due to these falls. She had no history of unusual infections earlier in her life, and there was no suggestion of immunodeficiency in her laboratory data. The C-reactive protein (a non-specific marker of systemic inflammation) was minimally elevated at 1.3 mg/dL. Her examination was normal with no neurological deficits. A CT scan of her lungs was normal. A radionuclide bone scan demonstrated increased uptake in the vertebral body of T9, and magnetic resonance imaging (MRI) showed a soft tissue mass anterior to the vertebral bodies of T8, T9, and T10. Thoracoscopic biopsy of the T9 vertebral body demonstrated necrotizing granulomas of the bone; special stains for bacteria, mycobacteria and fungi were negative (Figure 8). She was treated initially for both *M. tuberculosis* and NTM with isoniazid, rifampin, ethambutol and clarithromycin. After the culture grew only *M. abscessus*, she was treated with only clarithromycin; the other drugs for tuberculosis were discontinued. A follow-up MRI showed resolution of the edema and overall improvement at 4 months. The clarithromycin was discontinued after 6 months, and she was well with a normal C-reactive protein 3 months later. She was advised to discontinue roller-skating and other potentially traumatic activities and to be closely monitored for relapse.

### Discussion

Although most cases of disseminated NTM disease suggest the presence of an immunodeficiency, NTM infections can occasionally occur at distant sites from the lung in special situations. Obviously these organisms can gain access to bone or other internal structures during penetrating trauma. However, this case was associated with blunt trauma and so illustrates the principle of ‘locus minoris resistentiae,’ i.e., a place of less resistance, in this case to microbes. This unusual case was previously published along with two similar cases in 2001 (Chan et al., 2001). In retrospect, such a case now would probably be treated not only with a macrolide like clarithromycin, but also with at least one intravenous medication such as amikacin. The isolate in this case was most likely a *M. massiliense* subspecies that was susceptible to clarithromycin. Interestingly, shorter courses of therapy than are used for pulmonary disease are acceptable for bone infections.

## Nosocomial Infections With *Mycobacterium chimaera* After Cardiac Surgery

### Case

A 64-year-old man had cardiac surgery for a mitral valve porcine mitral valve replacement and a new prosthetic aortic valve in March of 2012. His postoperative course was uneventful until 5 months after the surgery when he developed persistent fevers between 103 and 104 degrees Fahrenheit. Blood cultures were negative, and transthoracic and transesophageal echocardiograms were normal. He was treated empirically with both oral and intravenous antibiotics for culture negative endocarditis in the setting of his recent surgery. In December 2012 (9 months after surgery) blood cultures drawn 30 days prior grew *M. chimaera (*a species in the *M. avium* complex*).* Despite optimal medical management with intravenous and oral multi-drug therapy, he remained persistently bacteremic for 2 years. Unfortunately, despite multi-drug therapy and close medical follow-up, the patient died of his persistent bacteremia.

### Discussion

A prolonged outbreak of *M. chimaera* infections after open-chest cardiac surgery was first reported by Swiss investigators in 2015. After the recognition of a cluster of cases of disseminated *M. chimaera* after cardiac surgery at the same hospital as our patient’s, the US Centers for Disease Control and Prevention (CDC) and the Pennsylvania Department of Health investigated the outbreak. They found that the heater-cooler devices (HCDs) used to maintain the patients’ body temperature during extracorporeal circulation (also known as cardiac bypass) were contaminated with *M. chimaera*, confirming similar findings by the Swiss investigators (Perkins et al., 2016). The isolates obtained from HCDs from three different European countries were nearly identical to those from the manufacturing site in another study. The results of these investigations strongly suggested a point-source contamination in the manufacturing of a specific HCD. There is a growing body of literature on this problem, and investigations are on-going.

Whole exome sequencing revealed that our patient had a mutation of interferon regulatory factor 8 (IRF8), which regulates expression of interferon-alpha and interferon beta genes. IRF8 is critically important in immune responses to intracellular pathogens, especially mycobacteria, involving the production of interleukin-12 in response to interferon-gamma (Hambleton et al., 2011). This mutation likely contributed to his susceptibility and death due to this infection.

## Conclusion

The cases presented in this paper demonstrate that NTM infections vary widely in presentation, treatment, and clinical outcome. While ubiquitous in the environment, NTM species tend to infect certain vulnerable patient populations, and these infections can often be organized into clinical phenotypes. While many NTM species have low pathogenicity, some opportunistic pathogens are important causes of human disease. Infection with these organisms, including MAC, MABSC, *M. kansasii*, *M. xenopi*, and others, are increasing in incidence and geographic distribution. Most human disease is pulmonary, primarily affecting immunocompetent patients with underlying structural lung disease such as bronchiectasis or COPD. Disseminated disease is usually more life-threatening, and primarily occurs in immunocompromised individuals. Among those most severely affected by disseminated disease are children with specific primary immunodeficiencies, and those with acquired T cell immunodeficiency due to anti-cytokine antibodies or iatrogenic administration of immunosuppressive medications, particularly tumor-necrosis factor alpha antagonists.

We hope that the host variability in NTM disease presented in this paper serves to stimulate and inspire basic and translational scientists to consider a similar variety of innovative interventions. For example, the poor response to treatment and increased mortality seen in fibrocavitary lung disease might be improved by drugs targeting biofilm dispersion (COPD Case). Inhaled liposomal amikacin was recently approved for pulmonary MAC disease in the United States, and translational studies on its role in biofilm infections may be a next step. Elucidation of the pathogenesis of non-CF bronchiectasis in tall, thin women may enable us to 1 day prevent the development of the disease. Candidate areas of research for the nodular-bronchiectasis MAC phenotype include leptin and ghrelin hormone influences on the immune response, cellular senescence, and the aging lung. In CF, research is needed on potential airborne transmission of *M. abscessus*, biofilm formation in the airways by *M. abscessus*, the effect of new CFTR modulator drugs on mycobacterial infection, among others. In PCD, it is possible that drugs improving the function of cilia may have a role in preventing NTM infections, and perhaps gene mutations could be corrected in the future. HP could be prevented by improved water disinfection techniques, and disseminated disease acquired during cardiac bypass could also be prevented by improved disinfection of heart-lung machines. Disseminated diseases associated with immunodeficiencies could be prevented by better immunomodulatory drugs that could correct such defects. Obviously, most patients would benefit from simpler, shorter and more effective drug treatment regimens against the mycobacteria, and the role of inhaled liposomal amikacin in such regimens continues to be defined.

The cases that we have presented illustrate the broad spectrum of NTM disease. We hope that these clinical vignettes have provided non-clinical scientists with a better understanding of human NTM diseases.

## Author Contributions

All authors contributed sections to the manuscript, and all reviewed and contributed to revisions.

## Conflict of Interest Statement

The authors declare that the research was conducted in the absence of any commercial or financial relationships that could be construed as a potential conflict of interest.
